# Effect of sperm DNA fragmentation on embryo development: clinical and biological aspects

**DOI:** 10.5935/1518-0557.20170061

**Published:** 2017

**Authors:** Cristian Alvarez Sedó, Melina Bilinski, Daniela Lorenzi, Heydy Uriondo, Felicitas Noblía, Valeria Longobucco, Estefanía Ventimiglia Lagar, Florencia Nodar

**Affiliations:** 1 Centro de Estudios en Genética y Reproducción (CEGYR), Buenos Aires, Argentina

**Keywords:** blastocyst, DNA fragmentation, blastulation rate

## Abstract

**Objective:**

The aim of this study was to investigate the effect of sperm DNA fragmentation on fertilization rate, embryo development (blastulation rate), and pregnancy outcomes for ICSI cycles performed in a cohort of couples using donor eggs and to assess the remaining embryos that were not transferred or frozen for apoptotic markers.

**Methods:**

Eighty-two women (egg recipients) were included in the study (2016) were included in the study. The recipients' mean age was 41.8±5.1 y/o (36-49), while the egg donors' mean age was 30.8±2.1 y/o (27-33). Even though donor egg cycles with frozen sperm samples are performed regularly in our center, 35 cycles were done using fresh sperm samples. The mean age of the males involved in the procedure was 40.1±5.2 y/o. Fertilization, blastulation, and pregnancy rates were assessed. The patients were divided into two groups, TUNEL <15% and ≥15%. In arrested embryos, ICC was performed to detect cleaved caspase-3, survivin, TUNEL, and DNA. The Student's *t*-test was used in between-group comparisons. The Mann-Whitney *U*-test was used to assess homogeneity. Pearson's correlation coefficient was also calculated. *p*<0.05 was considered statistically significant.

**Results:**

This study showed that there is a negative correlation (R=-0.5) between DNA fragmentation and blastulation rate. High levels of DNA fragmentation were associated with low blastulation and pregnancy rates (per transfer); however, fertilization rate was not affected. Samples with higher levels of DNA fragmentation were associated with higher levels of DNA fragmentation in blastomeres without activating the apoptotic pathway (9.1% vs. 15.9%) (*p*<0.05). Blastomeres from samples with high DNA fragmentation activated the apoptotic pathway in higher levels than samples with TUNEL <15% (16.4% vs. 21.9%) (*p*<0.05).

**Conclusion:**

Sperm DNA fragmentation was negatively correlated with blastulation and pregnancy rates even in good quality oocytes. High levels of DNA damage promote embryo arrest and induce the activation of the apoptotic pathway.

## INTRODUCTION

Male factor accounts for 30-40% of all cases of human infertility. In the past, medical decisions on treating infertile couples were based mostly on the results of conventional semen analysis, assessing sperm concentration, motility, and morphology in one or more semen samples.

In the early days of ART, severe male factor infertility yielded frustrating results, in the form of poor fertilization and pregnancy rates. With the preliminary reports on ICSI (intracytoplasmic sperm injection) in the early 1990s, clinicians and embryologists believed a solution had been found to all cases of male factor infertility. However, unsuccessful treatments despite the use of ICSI have indicated that other factors may be involved, including sperm DNA fragmentation or sperm morphologic damage undetected by the standard magnification used in conventional ICSI. More recently, several number of techniques designed to improve sperm selection for conventional ICSI have demonstrated to increase fertilization rates, enhance embryo quality after successful fertilization, and optimize pregnancy rates.

Several studies have demonstrated the importance of the stability of sperm nuclei and its correlation with successful reproduction in animals and humans, and the association of sperm nucleus damage with low fertilization rates, poor embryo implantation, and increased miscarriage rates (Aitken *et al.*, 1998; Morris *et al.*, 2002; Bungum *et al.*, 2004; Carrell *et al.*, 2003; Seli *et al.*, 2004; Virro *et al.*, 2004; Lewis & Aitken, 2005; Aitken *et al.*, 2009; Meseguer *et al.*, 2008; Zini & Sigman, 2009; De Iuliis *et al.*, 2009; Barratt *et al.*, 2010; Sakkas & Alvarez, 2010).

DNA damage may occur in the form of single or double strand breaks, and both types can be analyzed and/or quantified through different methods, including SCD (Sperm chromatin dispersion), SCSA (Sperm Chromatin Structure Assay) and TUNEL (terminal deoxynucleotidyl transferase dUTP nick end labeling) (Evenson *et al.*, 1980; Gorczyca *et al.*, 1993; Fernández *et al.*, 2005; Chohan *et al.*, 2006). DNA damage may have a negative impact in IVF-ICSI results (Evenson *et al.*, 2002; Cordelli *et al.*, 2005; Sergerie *et al.*, 2005; Greco *et al.*, 2005; Boe-Hansen *et al.*, 2006; Makhlouf & Niederberger, 2006; Avendaño *et al.*, 2009a; Avendaño *et al.*, 2009b; Góngora-Rodríguez & Fontanilla-Ramírez, 2010; Borini *et al.*, 2006; Ni *et al.*, 2014).

In the first days of embryo culture, morphological criteria alone are generally poor predictors of embryo development and ability to achieve pregnancy (Guerif *et al.*, 2010; Nel-Themaat & Nagy, 2011). However, embryos are still categorized and chosen for transfer based on morphological and developmental scores (Alpha Scientists in Reproductive Medicine and ESHRE Special Interest Group of Embryology, 2011).

Higher levels of DNA fragmentation (SDF >30%) in sperm cells have been associated with lower blastocyst formation rates (Virro *et al.*, 2004). Nasr-Esfahani *et al.* (2005) reported that embryos derived from spermatozoa with high levels of DNA damage are less likely to reach later developmental or blastocyst stages. However, in these studies the blastulation rates between the groups with SDF >30% and SDF <30% in IVF cycles were not different. The most likely explanation for this is “natural” selection during IVF. In that sense, it seems that ICSI results and embryo development (ICSI) are significantly affected by sperm quality.

Finally, regarding apoptosis, anomalies in cell death control have been implicated as a cause or contributing factor in a range of diseases, including cancer, autoimmunity, and degenerative disorders. Cell death control involves several proteins that promote or inhibit apoptosis and an evolutionarily conserved multistep cascade. A number of proteins, such as Bcl-2, Fas and Bax, affect processes upstream of the cascade. Survivin, an apoptosis inhibitor, may prolong cell survival by targeting terminal effector caspase-3. Located at the end of the cascade, caspase-3 acts as both an initiator and executor of the apoptotic process. Therefore, survivin and caspase-3 have received significant attention in the discussions on apoptosis (Li *et al.*, 2004).

This study looked into the effect of sperm DNA fragmentation on fertilization rates, embryo development (blastulation rate), and pregnancy outcomes of couples using donor eggs offered ICSI cycles. The remaining embryos that were not transferred or frozen were also assessed for apoptotic markers.

## MATERIALS AND METHODS

### Population

This study included 82 egg recipients submitted to ART procedures (2016). The recipient's mean age was 41.8±5.1 y/o (36-49) and the mean age of the oocyte donors was 30.8±2.1 y/o (27-33). Even though donor egg cycles with frozen sperm samples are performed regularly in our center, 35 cycles were done using fresh sperm samples. The mean age of the males involved in the procedure was 40.1±5.2 y/o.

Egg donors had to comply with the following requirements of the egg donation program: antral follicle count >16, negative serology, psychological and genetic counseling, and normal karyotype. A clinical geneticist tested the patients for relevant family history.

### Egg donor controlled ovarian stimulation

The donors were placed on a flexible GnRH antagonist protocol for ovarian stimulation, with daily doses of 225-300 IU of a gonadotropin (Menopur^®^) (Ferring) daily. When the leading follicle reached a diameter of 14 mm, GnRH antagonist Orgalutran (MSD) was administered daily until the day of Lupron^®^ injection. Once the leading follicle reached 17-18 mm in diameter and estradiol levels were >+500 pg/ml, leuprolide acetate (Lupron^®^, 2 mg) was administered 36 hours prior to oocyte retrieval. Then the IVF procedure (ICSI) was performed.

### Endometrial preparation for ET

Seventy-five embryo transfers (blastocyst) were performed; patients received oral estradiol Valerate (Ronfase^®^) 4 mg daily from day 2 of the menstrual cycle. On day 10, an ultrasound examination was performed. After ultrasound confirmation of endometrial thickness >6mm and absence of ovarian activity, progesterone (Utrogestan^®^) 600 mg daily was added for 5 days before ET and up to 14 weeks afterwards when pregnancy was confirmed.

The dose of Ronfase was increased to 6 mg daily if endometrial thickness was less than 6mm. Ultrasound examination was repeated within 7 days and cycles were cancelled if the endometrium failed to reach the minimum thickness.

### Source of embryos

All remaining human embryos were donated for research with the consent of the couples submitted to ART procedures at CEGyR (Buenos Aires, Argentina). The Internal Review Board and Ethics Committee of CEGyR approved the procedures involving human embryos. None of the presumed embryos donated for this project were transferred to recipients after ICSI. If they had not been donated to this research, these embryos would have been discarded. All embryos were fixed immediately 5 days after oocyte fertilization was confirmed.

### Chemicals and antibodies

All chemicals were obtained from Sigma Chemical Co. (St. Louis, MO, USA), unless stated otherwise. Cleaved caspase-3 (CC3) and Survivin (Surv) were detected using anti-full-length human CC3 (rabbit monoclonal, dil: 1:100, Cell Signaling, USA), and Surv (mouse monoclonal, dil: 1:100, Novus Biologicals, USA). Alexa Fluor secondary antibodies were obtained from Molecular Probes (Invitrogen, US). TUNEL (Roche, USA) assays were also performed to detect DNA fragmentation. Hoechst 33258 (Sigma) was used for DNA staining.

### Embryo processing

One hundred and eighty-seven embryos from 82 patients were collected after ICSI and studied. All embryos were individually processed. The zona pellucida was slightly dissolved by incubation in acidic Tyrode´s solution (Irvine Scientific, US), and then the embryos were fixed and processed by immunocytochemistry (ICC) (see below).

### Detection of cell damage and apoptosis in embryos by immunohistochemistry

**Figure 1:**
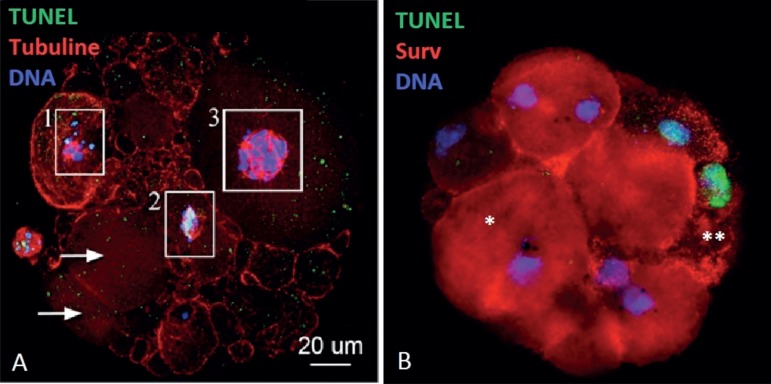
In A, ICC for TUNEL and DNA staining fragmented DNA (1 and 2), non-fragmented DNA (3), and some blastomeres without DNA (arrows). In B, Blastomeres without DNA damage and Surv (+) (*) or DNA fragmentation and Surv (-) (**).

**Figure 2:**
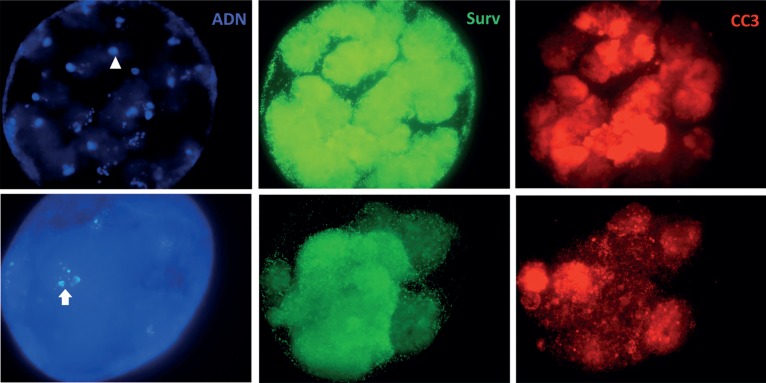
Embryos with condensed DNA (arrowhead) and TUNEL (+) (arrow). Surv and CC3 show different positive staining.

The human embryos were fixed for 45 min in 2% formaldehyde and washed with PBS + 0.1% Triton X-100 for an additional 45 min (method modified and based on Messinger and Albertini, 1991). After fixation and washing, the samples were blocked for at least 1 h in PBS + 0.3% bovine serum albumin (BSA) + 1% fetal calf serum prior to incubation in humidified chambers with primary and secondary antibodies overnight at 4°C and for 2 hours at 37°C, respectively. The embryos were washed several times with PBS-BSA. After washing, they were incubated in TUNEL solution for 1 hour at 37°C. Finally, the embryos were incubated with Hoechst 33258 for DNA detection. Some images were obtained using an Olympus spectral confocal microscope, with laser lines at 488-, 568- and 633 -nm wavelengths and then processed using Adobe Photoshop C5; additional images were captured with an Olympus BX40 Fluorescence Microscope. Negative controls were run in the absence of primary antibodies. This assay allowed us to determine the cytoplasmic activation of CC3 in the blastomeres of each of the embryos (apoptosis); Surv activation meant that the cell performed a strategy to stop the cell death; TUNEL (positive) meant that DNA fragmentation (damage) had occurred, but nuclear condensation and TUNEL were the final evidence of cell death by apoptosis (Figures 1 and 2).

### Sperm analysis and DNA fragmentation

**Figure 3:**
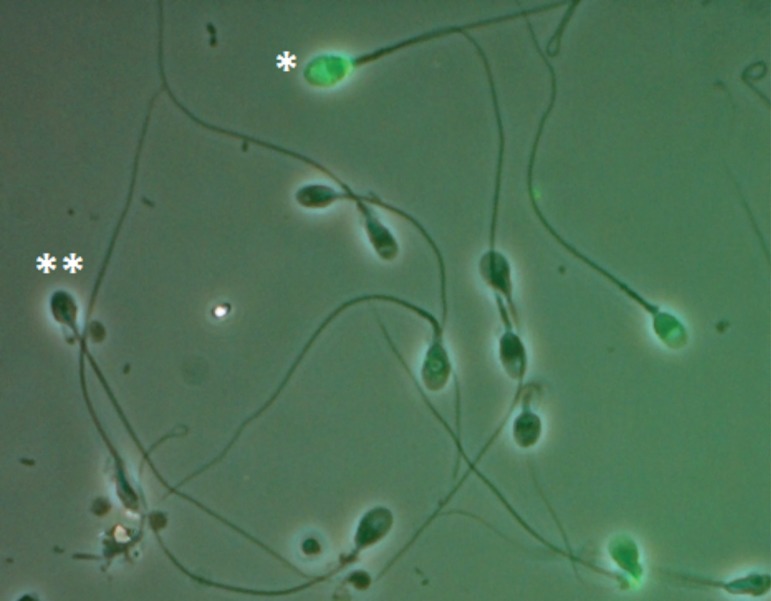
TUNEL assay to assess sperm DNA fragmentation. Positive cells (*), negative cells (**).

Semen analysis was performed accordingly to the procedure established by the WHO (2010). After motile sperm isolation, the samples were fixed in 2% formaldehyde in phosphate-buffered saline solution (PBS; pH 7.4) for at least 1 hour. Each sample was placed into one well of a multiwell (4-mm diameter) Teflon-printed slide (Electron Microscopy Sciences) and allowed to settle. After 2-3 hours, each well was washed with 1X PBS (three times, 5 minutes each); the cells were then permeabilized with cold methanol. Before incubation with TUNEL solution, each well was washed again with 1X PBS. For each sample, one extra well was incubated with DNAse (1 U/mL; Sigma) for 30 minutes at 37°C as a positive control, and in another well the TUNEL ''enzyme' 'solution was omitted as a negative control. Then all samples were incubated in TUNEL solution (Roche,) for 1 hour at 37°C. All samples were finally washed with 1X PBS (three times, 5 minutes each), and mounted in Vectashield H-1000 medium (Vector Laboratories). 500 spermatozoa were counted by fluorescence microscopy. TUNEL staining was evaluated on a fluorescence microscope using a green filter (fluorescein isothiocyanate, 488 nm) (Figure 3).

### Statistics

The Student's *t*-test was used for between-group comparisons. The Mann-Whitney *U*-test was used to assess homogeneity. Pearson's correlation coefficient was also calculated. *p*<0.05 was considered statistically significant. Statistical analyses were carried out on software program MedCalc 12.5 (Belgium).

## RESULTS

**Figure 4:**
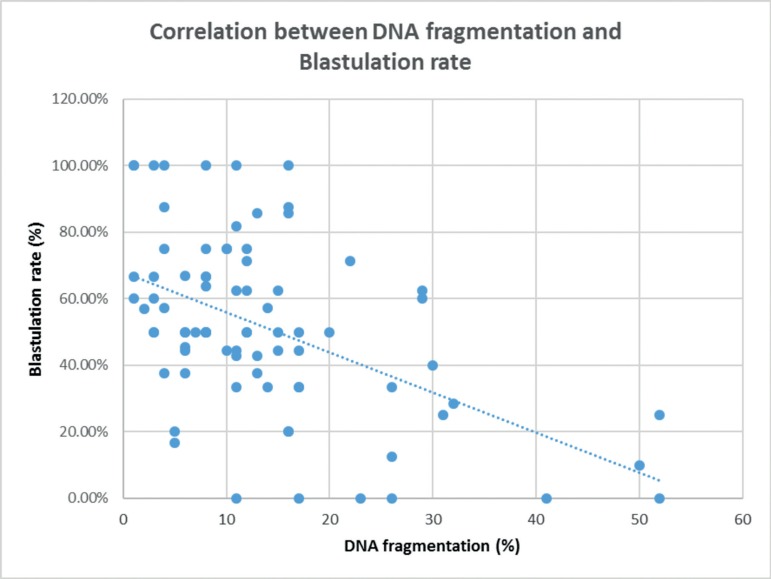
Correlation analysis between DNA fragmentation and Blastulation rate (R=-0.5).

Table 1 shows sperm analysis data. Clinical outcomes (ICSI results) in terms of fertilization, blastulation, and pregnancy rates are shown in Table 2. The same data is shown in Table 3 for sperm samples with high (≥15) or low (<15%) levels of DNA fragmentation. The results revealed the existence of a negative correlation (R=-0.5) between DNA fragmentation and blastulation rates (Fig. 4), and an association between high levels of DNA fragmentation and low blastulation and pregnancy rates (per transfer); fertilization rate was not affected.

**Table 1:** Results of sperm analysis performed according to the WHO guidelines (2010).

Sperm parameters (%)	N=82
**Volume (mL)**	2.7±0.8
**Sperm Concentration (mill/mL)**	68.5±43.1
**Sperm Morphology using Kruger’s strict criteria**	6.5±3.8
**Sperm Progressive Motility (a+b)**	50.8±16.9
**PMN**	0.96±1.64
**DNA fragmentation (TUNEL)**	13.5±11.1

**Table 2:** Clinical outcomes for the studied population.

Clinical outcomes	X±SD	Min-Max
**Female age**	41.8±5.2	36-49
**Donor age**	30.8±2.1	27-33
**Male age**	40.1±5.2	33-60
**Nº of oocytes assigned (MII)**	8.7±2.1	4-14
**Fertilization rate (%)**	75.4±18.9	20-100
**Blastulation rate (%)**	51.8±26.3	0-100
**Pregnancy rate (%)/transfer**	50/75 67%	

**Table 3:** Clinical outcomes - Comparison between patients with TUNEL <15% vs. ≥15%.

	DNA fragmentation <15%	DNA fragmentation ≥15 %	*p*
**N**	54	28	
**Nº of oocytes assigned (MII)**	8.9±2.3	8.5±1.8	0.75
**Strict morphology (%)**	6.5±3.1	5.9±4.2	0.40
**DNA fragmentation (%)**	7.6±3.8	24.9±11.5	**0.001**
**Fertilization rate (%)**	76.1±19.4	73.8±18.2	0.62
**Blastulation rate (%)**	59.2±22	37.5±28	**0.003**
**Pregnancy rate (%)/transfer**	38/51 74.5%	12/24 50%	**0.06** ****

On the other hand, when the remaining embryos were analyzed, samples with higher levels of DNA fragmentation were observed to induce higher levels of DNA fragmentation in blastomeres without activating the apoptosis pathway (9.1% vs. 15.9%) (*p*<0.05). Likewise, blastomeres from samples with high DNA fragmentation activated the apoptotic pathway in higher levels than TUNEL <15% (16.4% vs. 21.9%) (*p*<0.05). The final expression of cell death, TUNEL (+), Surv (+), CC3 (+) and DNA condensation, was increased in embryos coming from sperm samples with high levels of DNA damage (3.0% vs. 8.8%) (*p*<0.05). Finally, when TUNEL and CC3 were positive and Surv was negative, there were no statistical differences between groups (Table 4).

**Table 4:** Blastomere analysis - Comparison between patients with TUNEL <15% vs. ≥15%.

	DNA fragmentation <15%	DNA fragmentation ≥15 %	*p*
**Nº embryos**	112	75	
**Nº of Blastomeres assessed **	860	432	
**Blastomeres without DNA damage**	458 (53.2%)	137 (31.7)	**0.0001**
**Blastomeres with DNA damage** **CC3 (-) Surv (-)**	78 (9.1%)	69 (15.9%)	**0.0004**
**Blastomeres with DNA damage ** **CC3 (+) Surv (-)**	132 (15.3%)	76 (17.6%)	0.29
**Blastomeres without DNA damage** **CC3 (+) Surv (+)**	25 (2.9%)	17 (3.9%)	0.45
**Blastomeres with DNA damage ** **CC3 (+) Surv (+)**	141 (16.4%)	95 (21.9%)	**0.007**
**Blastomeres with DNA damage ** **CC3 (+) Surv (+) Nuclear condensation**	26 (3.0%)	38 (8.8%)	**0.0001**

## DISCUSSION

Semen quality is usually expressed in terms of sperm concentration, motility, and morphology (WHO, 2010). Our group previously reported that these parameters, specifically morphology and motility, were closely related to DNA alterations (Uriondo *et al.*, 2011, Alvarez Sedó *et al.*, 2012). Though sperm DNA damage is not considered in regular sperm analysis, the literature suggests that sperm DNA damage produces relevant impact on male fertility. Various theories have been put forward to explain sperm DNA damage (apoptosis, chromatin remodeling, oxidative damage) (Sakkas & Alvarez, 2010).

During the course of natural selection, effective conception can only occur following the fertilization of an oocyte by sperm with intact DNA. However, assisted reproductive technologies have increased the possibility of anomalous spermatozoa being used to fertilize oocytes (Tavukçuoğlu *et al.*, 2012). Sperm DNA fragmentation is an important parameter of sperm quality that can be used to assess sperm nuclear integrity, which itself plays an important role in fertilization and embryo development (Bungum *et al.*, 2007). The role of male factor infertility on embryo development has gained attention since the introduction of ICSI as a treatment option for patients with very poor sperm characteristics. Our study demonstrated that blastulation rates were associated with high levels of DNA damage, suggesting a very early onset of paternal effects on embryo development.

In the present study, sperm DNA damage was assessed by TUNEL assay, performed on motile sperm prepared with a swim-up procedure and used for ICSI. Our group previously demonstrated that the predictive ability of the sperm DNA integrity test, performed on raw sample, diminished when spermatozoa were prepared using techniques such as Swim-up or centrifugation density gradient (Rougier *et al.*, 2013, Alvarez Sedó *et al.*, 2013). However, in our population, several samples had high levels of DNA fragmentation even after motile sperm isolation.

This preliminary data found no relationship between sperm DNA damage and fertilization rates in ICSI (*p*=0.62), but the blastulation rate was clearly diminished when DNA damage was high (*p*<0.05). On the other hand, patients with low DNA fragmentation (<15%) had a clear tendency to attaining higher pregnancy rates (*p*=0.06). These results may be accounted for by the fact that high DNA fragmentation probably does not impede fertilization, but prevents blastulation and/or successful embryo development (Ahmadi & Ng, 1999). However, this issue was mostly observed in ICSI patients; when IVF was performed, these differences were not evinced, probably due to "natural" selection of sperm by the oocyte (Borini *et al.*, 2006). The amount of sperm DNA damage was related to embryo development to the blastocyst stage, a time when the embryonic genome is activated, transcriptional activity has begun, and the paternal genome plays a significant role in embryo function toward implantation (Seli *et al.*, 2004).

This is the first report that used an egg donation model to assess the impact of DNA damage over clinical and biological outcomes. Our biological results showed that sperm DNA damage might promote blastomere DNA fragmentation without the activation of the apoptotic machinery, probably due the injection of sperm with slight DNA damage that was able to advance during the embryonic development. However, other mechanisms might be at play in embryo arrest. On the other hand, most of the blastomeres showed a complete apoptotic pattern (TUNEL (+), CC3 (+) and Surv (+)), revealing that good quality oocytes respond adequately to the induction of apoptosis. However, this was observed largely in embryos that came from samples with high levels of DNA damage, confirming that sperm damage can cause further arrest in embryonic development.

In conclusion, our data indicated that sperm DNA fragmentation significantly affected embryo blastulation and implantation in ICSI patients who received donated eggs. More specifically, this study showed, for the first time, that sperm DNA fragmentation might compromise the progression of embryo development, resulting in arrested embryos. This study also underlined the better predictive value of DNA fragmentation analysis versus traditional sperm parameter evaluation in the assessment of ART outcomes. For this reason, sperm DNA fragmentation should be considered during the assessment of semen quality.

## CONCLUSIONS

Sperm DNA fragmentation had a negative correlation with blastulation and pregnancy rates even with good quality oocytes. High DNA damage levels promoted embryo arrest and induced the activation of the apoptotic machinery.
